# Demographic and Ecological Survey of Dog Population in Aba, Abia State, Nigeria

**DOI:** 10.1155/2014/806849

**Published:** 2014-04-09

**Authors:** Gbeminiyi Richard Otolorin, Jarlath U. Umoh, Asabe Adamu Dzikwi

**Affiliations:** Department of Veterinary Public Health and Preventive Medicine, Faculty of Veterinary Medicine, Ahmadu Bello University, Zaria, Kaduna State, Nigeria

## Abstract

Dog ecology is essential in understanding the distribution, structure, and population density of dogs and pattern of dog ownership in any given area. A cross-sectional study was designed to study dog ecology in Aba, Abia state, Nigeria, from April to June 2013. The study revealed that the 500 households surveyed possessed 5,823 individuals and 747 dogs, giving a dog to human ratio of 1 : 7.8; hence dog population in Aba was estimated to be 68,121. About 495/747 (66.3%) of the dogs were exotic and 465/747 (62.2%) were males. A total of 319/500 (63.8%) of the households had fences that restrained dog movement and there was no incidence of dog bite in 447/500 (89.4%) of the households surveyed. There were statistical associations between vaccination against antirabies and breeds of dogs (*χ* ^2^ = 79.8, df = 2, *P* < 0.005). Exotic breed (adjusted OR = 0.39; CI = 0.23–0.65) and local breed of dogs (adjusted OR = 0.08; CI = 0.04–0.14) had less odds of being vaccinated as compared to crossbreed of dogs. About 126 dogs (2.5 dogs per street) were estimated from street counts survey. The relative high dog to human ratio and low vaccination coverage of owned dogs population pose public health concerns requiring adequate public health education and proper antirabies vaccination coverage of dogs in the study area.

## 1. Introduction

Dog ecology involves studies on dog population density, dog population structure, and pattern of dog ownership [1]. In nearly all parts of the world, dogs pose serious human health, socioeconomic, political, and animal welfare problems [2]. In developing countries, the domestic dog is the most important reservoir and vector of human rabies, accounting for 99% of exposures (WHO, 1992). Rates of disease transmission depend on the density of the dog population and social behaviour that determines the extent of contact [3]. As recognised by World Health Organization [4] dog demography studies are key to addressing many of these knowledge gaps even more so as rapid changes in human and dog demographics have important implications for the dynamics and control of rabies [5].

Rabies epidemiology in the dog reservoir is directly associated with dog ecology; thus, better understanding of dog ecology would be useful for designing appropriate rabies control measures in the dog population [6]. The dog : human ratio most commonly lies between 1 : 6 and 1 : 10, but considerable variation exists [7]. Urban areas in Africa have a ratio of 1 : 21.2, while rural areas in Africa have a ratio of 1 : 7.4 [8]. The ratio of owned dogs to people is usually higher in rural areas of a country, but there is also considerable variation within cities [9]. The structure and turnover of a dog population are determined by a great number of different factors. Its analysis depends on vital statistics such as sex and age ratios, natality and rearing success, and survival and mortality rates [10]. Some dog population densities studies in Nigeria have been carried out applying techniques such as total street-dog count and estimates from the rate of capture and recapture [11–14]. This research aims to determine the distribution, structure, and population density of dogs and the pattern of dog ownership (dog ecology) in Aba, Abia state, Nigeria.

## 2. Materials and Methods

### 2.1. Study Area

The study was carried out in Aba, Abia state, located in Southeastern Nigeria, consisting of two local government areas, Aba North and Aba South. Aba is a major urban settlement and commercial centre in a region that is surrounded by small villages and towns. Aba is well known for its industrial nature, manufacturing industries, and large scale trading. Aba lies between latitudes 5°07′N and between longitudes 7°22′E. With a temperature of about 21°C, the climate is humid tropical type and is characterized by wet and dry seasons [15].

### 2.2. Study Design

A cross-sectional study was carried out to evaluate dog ecology in Aba, Abia state, between April and June 2013 in Aba North and Aba South local government areas (LGAs).

### 2.3. Survey Methods

Fifty (50) streets in the study area consisting of twenty-five (25) streets each in Aba North and Aba South LGAs were surveyed using* compound questionnaire survey* and* street counts*. A total of 500 households, 250 each in Aba North and Aba South local government area, were surveyed.

#### 2.3.1. Compound Questionnaire Survey of Dogs

Starting from the 1st major street of each local government area, every 5th street was surveyed using systematic random sampling technique. In each chosen street, starting from the 1st houses on either side, every 10th house was selected, and an adult member on either side was interviewed using questionnaire. Information obtained included household information such as numbers of dogs in the premises, housing and control of dog movement, and history of dog bites and individual dog information such as breed, sex, age, source of dog, source of food, and vaccination status. A total of ten (10) houses were surveyed in each street chosen. Houses without dogs or houses that refused to participate in the compound questionnaire survey were skipped for the next house possessing dog(s).

#### 2.3.2. Street Counts

Dogs on the street were counted using the photographic recapture technique (Beck's method). The area surveyed in Aba North and Aba South LGAs was divided into 4 areas determined by the major roads (Faulks road in Aba North, Okigwe road in Aba North, Asa road Aba south in Aba South, and Port harcourt road in Aba South) linking the streets. It involved two street counts on two different days and the counts were averaged to reduce sampling error. On each day of the study, the counting of dogs was carried out early in the morning (between 6 and 9 am) and in the evening (5.30 to 7.00 pm). Every dog within a given distance was photographed and recorded. The number of dogs counted in the selected streets in each area was estimated using the following formula:

(1) $$\begin{array}{c} N=\frac{\sum \left(Mn\right)}{\sum m}, \end{array}$$

where *M* = the number of dogs photographed each time and considered “marked,” that is, “observed”; *m* = the number of dogs recognised as being previously photographed, that is, “reobserved”; ∑*m* = the summation of *m* to that point in time; *n* = the total number of dogs previously observed; that is, each day's observations (*M*) less than those previously observed (*m*) would be added to each day; *Mn* = the product of each days *M* and *n*; ∑(*Mn*) = the summation of *Mn* to that point in time; and *N* = the population estimate [10].

### 2.4. Data Analysis

In this study, data generated was analysed using the statistical packages for social sciences (SPSS) Version 17.0. Data obtained was presented using tables and charts. Chi-square test was used where appropriate to test for association of variables obtained from the questionnaires. Values of *P* < 0.05 were considered significant.Odds ratio and adjusted odds ratio using multivariable logistic regression analysis were also utilized.

## 3. Results

In the 500 households surveyed 63.8% had fences/walls that completely restrained dogs movement and 23% households had fences that do not restrain dogs movement, while 13.2% of the households had no fence. Most of the households (73.6%) had specially constructed houses/cages as housing for their dogs, while 5.4% indicated their dogs were kept anywhere on the premises. About 92.8% of the households never allowed their dogs to leave their premises, while 7.2% allow their dogs to roam freely in the neighbourhood (Table 1).

A total of 747 dogs were present in the 500 households surveyed. Majority of the dogs (66.3%) were exotic, 19% were local, and 14.7% were crossbreeds. Most of the dogs (62.2%) were males, while females were 37.8% of the total number of dogs. A total of 38.0% of the dogs were within 0 to 6 months of age and 39.4% greater than 12 months of age. During the day 58.6% of the dogs were confined to restrict their movement, while 1.7% of the dogs were confined during the night. A total of 47.9% of the dogs were vaccinated against rabies; only dogs that had attained the age of vaccination against rabies were considered in estimating the antirabies vaccination coverage in the 500 households surveyed (Table 2).

Among the 500 households surveyed there were 5,823 occupants and 747 dogs. The average number of dogs per each house surveyed was 1.5, while the dog to human ratio was 1 : 7.8. The street count of dogs was 126 dogs (67 dogs in Aba North and 59 dogs in Aba South). From the 2006 population census figure of Aba North and Aba South which is estimated as 531, 340 people the estimated dog population in Aba North and Aba South is about 68,121 (Table 3).

Chi-square analysis was used to determine association between dog vaccination status and various pieces of individual dog information. There were associations between breeds of dogs (*χ* ^2^ = 79.8, df = 2, *P* < 0.005), age of dogs (*χ* ^2^ = 22.9, *df* = 2, *P* < 0.005), reason for dog ownership (*χ* ^2^ = 8.1, df = 2, *P* < 0.005), confinement of dogs (*χ* ^2^ = 10.0, df = 3, *P* < 0.005), and rabies vaccination status of dog. These variables were further analysed using multivariable logistic regression analysis; exotic breed (adjusted OR = 0.39; CI = 0.23–0.65) and local breed of dogs (adjusted OR = 0.08; CI = 0.04–0.14) had less odds of being vaccinated as compared to crossbreeds. Similarly dogs used as guard dogs (adjusted OR = 0.65; CI = 0.37–1.12) and dogs used for breeding/sale (adjusted OR = 0.41; CI = 0.22–0.79) purposes had less odds of being vaccinated as compared to dogs used as pets (Table 4).

## 4. Discussion

Information obtained from 500 households surveyed indicates a relative high density of dogs in the study area (dog to human ratio of 1 : 7.8) probably due to the absence of religious and cultural inhibition to the keeping of dogs in Aba, Abia state. This is in contrast to the low density of dogs in other urban cities in Nigeria, where dog to human ratio is 1 : 43 in Makurdi, Benue state [16], 1 : 21 in Lagos, Lagos state, and 1 : 1000 in the moslem dominated part of Kaduna North, Kaduna state [7].

Most of the houses had fences that completely restrain dog movement as the survey was conducted in an urban area where most houses are properly demarcated from each other by well defined fences. Majority of the households had specially constructed houses/cages for their dogs and never allowed their dogs to leave the household premises. Dogs owned by inhabitants of the town are mostly exotic breed; hence they are well cared for because of the value attached to these dogs. Most of the respondents interviewed indicated low activity of free roaming dogs in their streets, with majority of the households reporting that no stray dogs eat in their homes. This is further confirmed by the result of the street count where few dogs (126) were seen in the 50 streets surveyed in the study area. Hence nuisances caused by free roaming dogs pose little problem in the area.

The antirabies vaccination coverage of dogs in the houses surveyed was estimated at 47.9%. This still falls below the World Health Organization standard of vaccination of 70–80% dog population in an area to boost herd immunity [17]. This vaccination rate can be further improved if there are more veterinary clinics in the city and dog owners who are mainly traders create time out of their busy schedules to give their dogs the required veterinary care. Local breed of dogs was the least vaccinated against rabies due to the low priority given to this breed by most owners as compared with exotic and crossbreeds. Dogs within 7–12-months-old had higher antirabies vaccination frequency; this may be because dog owners see dogs at this age category as more likely to be aggressive and likely to be involved in dog bites; also more male dogs were kept than female dogs as they are perceived as better guard dogs than the females and are hence kept for security purposes.

## 5. Conclusion

The study revealed that the relatively high dog to human ratio and the vaccination coverage which falls below the recommended WHO standard pose public health concerns and require proper and well planned intervention aimed at adequate public health education and adequate antirabies vaccination coverage of dogs in the study area. Also dog ecology survey was able to determine that 500 households possess an average of 1.5 dogs per household with an estimated dog population in Aba being 68,121. This information is very important and can serve as a guide in the planning of antirabies campaign programme in Aba, Abia state.

## Figures and Tables

**Table 1:** Household information on dog ecology obtained from respondents in 500 households in Aba North and Aba South LGA of Abia state (from April to June 2013).

Variable	Frequency	Percentage (%)
Enclosure of home		
No fence	66	13.2
Fence/wall, but does not restrain dogs	115	23.0
Fence/wall, completely restrain dogs	319	63.8
Housing of dogs		
Specially constructed house/cage	368	73.6
On house passage way/corridor	105	21.0
Anywhere on the premises	27	5.4
Control of dog(s) movement		
Never allowed to leave premises	464	92.8
Allow to roam freely	36	7.2
Do you observe stray dogs on your street		
Yes	112	22.4
No	388	77.6
Has any member of the household been bitten by stray dog in the last 12 months		
Yes	63	12.6
No	437	87.4
Do dogs other than yours eat at your home		
Yes	29	5.8
No	471	94.2
Total	**500**	**100**

**Table 2:** Information obtained from 500 dog owning households in Aba North and Aba South LGA of Abia state (from April to June 2013).

Variable	Frequency	Percentage (%)
Breeds of dogs		
Exotic	495	66.3
Local	142	19.0
Cross	110	14.7
Sex of dogs		
Male	465	62.2
Female	282	37.8
Ages of dogs		
0–6 month	284	38.0
7–12 month	169	22.6
>12 month	294	39.4
Source of dogs		
From own bitch	367	49.1
Bought	334	44.7
Received as gift	46	6.2
Reason for dog ownership		
Guard dogs	474	63.5
Sale/breeding	182	24.4
Pet	91	12.2
Confinement of dogs		
Day	438	58.6
Night	13	1.7
Day and night	260	34.8
Allow to roam	36	4.8
Feeding of dogs		
Household members	649	86.9
Neighbours	1	0.1
Find its own food	3	0.4
Neighbours/household	94	12.6
Rabies vaccination status		
Yes	298	39.9
No	324	43.4
Not up to vaccination age	125	16.7
Total	**747**	**100**

**Table 3:** Structure of dogs and human population in compound count carried out in Aba North and Aba South LGA.

Variables	Values
Number of compounds surveyed	500
Total number of people in the streets surveyed	**5823**
Total number of dogs	**747**
Dog human ratio	1 : 7.8
Average number of dogs per house	1.5
Total dog population size estimated from street count	**126**
2006 population census of Aba North and Aba South LGA	531, 340
Estimated dog population in Aba North and Aba South LGA	68, 121

**Table 4:** Chi-square and multivariable logistic regression analysis of vaccination status with individual dog information in Aba, Abia state.

	Vaccination status						
Variable	Yes	No	χ^2^	*P* value	Crude OR (95% CI on OR)	Adjusted OR (95% CI on OR) vaccinated dogs	Adjusted OR (95% CI on OR) unvaccinated dogs
Breeds of dogs							
Exotic	200	182	79.8	0.000	0.42 (0.26–0.68)	2.57 (1.54–4.28)	0.39 (0.23–0.65)
Local	23	113			0.08 (0.04–0.15)	13.30 (7.00–25.27)	0.08 (0.04–0.14)
Cross	75	29			1	1	1
Age of dogs							
0–6 months	82	77	22.9	0.000	1.71 (1.16–2.52)	0.75 (0.48–1.17)	1.34 (0.86–2.09)
7–12 months	103	66			2.50 (1.70–3.69)	0.40 (0.25–0.61)	2.55 (1.65–3.94)
	113	181			1	1	1
Reason for dog ownership							
Guard dogs	212	224	8.1	0.018	0.67 (0.40–1.10)	1.55 (0.89–2.69)	0.65 (0.37–1.12)
Sale/breeding	42	69			0.42 (0.24–0.78)	2.43 (1.27–4.65)	0.41 (0.22–0.79)
Pet	44	31			1	1	1
Confinement of dog							
Day	203	192	10.0	0.018	2.91 (1.33–6.45)	0.43 (0.19–1.02)	2.30 (0.98–5.42)
Night	7	5			3.89 (0.98–15.42)	0.35 (0.08–1.55)	2.86 (0.65–12.70)
Day and night	79	102			2.15 (0.95–4.87)	0.55 (0.23–1.34)	1.82 (0.75–4.42)
Allow to roam	9	25			1	1	1
Sex of dogs							
Male	188	193	0.81	0.368	1.16 (0.84–1.60)		
Female	110	131			1		
Source of dog							
From own bitch	123	160	4.5	0.104	0.59 (0.30–1.17)		
Bought	153	14			0.80 (0.41–1.58)		
Received as gift	22	17			1		
Total	**298**	**324**					
